# Sensitivity of *Deinococcus grandis rodZ* deletion mutant to calcium ions results in enhanced spheroplast size

**DOI:** 10.3934/microbiol.2019.2.176

**Published:** 2019-06-26

**Authors:** Yusuke Morita, Mai Okumura, Issay Narumi, Hiromi Nishida

**Affiliations:** 1 Biotechnology Research Center and Department of Biotechnology, Toyama Prefectural University, 5180 Kurokawa, Imizu, Toyama 939-0398, Japan; 2 Radiation Microbiology Laboratory, Department of Life Sciences, Faculty of Life Sciences, Toyo University, 1-1-1 Izumino, Itakura, Gunma 374-0193, Japan

**Keywords:** cell size, cytoplasm size, *Deinococcus grandis*, rod shape, *rodZ* deletion mutant, sensitivity to calcium ion, spherical shape, spheroplast enlargement

## Abstract

RodZ is a cytoskeletal protein associated with bacterial cell shape. It is a transmembrane protein located on the plasma membrane, and it binds to another cytoskeletal protein MreB. *Deinococcus grandis* contains a *rodZ* homolog. Although *D. grandis* is rod-shaped, it becomes spherical in shape when the *rodZ* homolog is disrupted. The *rodZ* deletion mutant was treated with lysozyme to generate spheroplasts. The spheroplasts enlarged in medium containing calcium chloride and penicillin. The *rodZ* deletion mutant spheroplasts were more sensitive to calcium ions than wild type. Cell and cytoplasm sizes of enlarged spheroplasts of the *rodZ* deletion mutant tended to be larger than those of wild type. Thus, disruption of *rodZ* enhances plasma and outer membrane expansion in *D. grandis* spheroplasts.

## 1. Introduction

The genus *Deinococcus* consists of both bacilli and cocci species [1]. *Deinococcus* lacks lipopolysaccharides on its outer membrane [2],[3]. However, the inner and outer membranes are structurally different [4],[5]. *Deinococcus grandis* is an aerobic Gram-negative, non-spore-forming, radioresistant, rod-shaped bacterium [6]. The spheroplasts of *D. grandis* enlarge in incubation medium containing penicillin under osmotically protective conditions [7]. The enlargement requires calcium or magnesium ions [7]. Outer membrane fusion occurs in the presence of calcium ions [8],[9]. Transcription or translation inhibitors prevents the spheroplast enlargement [7].

The amount and speed of outer membrane biosynthesis of *D. grandis* spheroplasts is higher than those of the plasma membrane [7]. Thus, enlarged spheroplasts have a large periplasmic space. Lipidomics of total cells showed that *D. grandis* has a unique lipid composition, as *D. grandis* lacks phosphatidylethanolamine (PE) and phosphatidylglycerol (PG), which are commonly found in other bacteria [7]. This is consistent with the fact that *Deinococcus radiodurans* has glycolipids and glycophospholipids but lacks PE and PG due to the lack of biosynthetic genes [10]–[12]. Lipid composition changes during enlargement of *D. grandis* spheroplasts [7]. This suggests that regulation of biosynthesis of the outer and plasma membranes may change during enlargement. Outer membrane components are synthesized in the cytoplasm or the inner leaflet of the plasma membrane. Following synthesis, these components are transported across the plasma membrane and through the periplasm to the outer membrane for assembly [13]. Thus, the transport system on the plasma membrane influences outer membrane biosynthesis.

RodZ is a bacterial protein associated with rod shape. The N-terminal region of RodZ is located in the cytoplasm, while the C-terminal region is located in the periplasm [14],[15]. The *rodZ* deletion mutant of *Escherichia coli* is not rod-shaped but round or oval [14],[15]. Homologs of *rodZ* are present in a wide range of bacteria [16]. RodZ binds to MreB, which is required for cell shape maintenance in rod-shaped bacteria [17]. MreB functions as the bacterial actin cytoskeleton [18],[19]. Thus, *rodZ* deletion mutants inhibit rod-shape formation by preventing the binding of MreB to RodZ.

*D. grandis* contains homologs of MreB (WP_058976727) and RodZ (WP_058975389) [1]. Lysozyme-induced enlarged spheroplasts have a large periplasmic space. Thus, plasma membrane expansion is limited. In the current study, in order to elucidate whether the MreB-RodZ cytoskeleton system inhibits plasma membrane expansion, we disrupted the *rodZ* homolog in *D. grandis*. The *rodZ* deletion mutant (Δ*rodZ*) was treated with lysozyme to induce spheroplast enlargement. We compared enlargement levels and morphology of Δ*rodZ* with those of the wild type.

## 2. Methods

### 2.1. Preparation and cultivation of spheroplasts

A single colony of *D. grandis* KS 0485 (ATCC 43672) was streaked onto a tryptone glucose yeast extract (TGY) agar plate (5 g/L tryptone [BD, Franklin Lakes, NJ], 1 g/L glucose, 3 g/L yeast extract [BD] and 15 g/L Bacto agar [BD]) and incubated for 2 to 3 d at 30 °C. A single colony was inoculated for primary culture followed by secondary culture in 10 ml of TGY broth. The colony was incubated at 30 °C with shaking, until It reached OD_600_ of 0.7. Cells (6 ml) were harvested via centrifugation at 7,000 rpm (6,684 × *g*) for 5 min. The supernatant was discarded, and the cells were washed once with 6 mL of PS buffer (4.56 g/L KH_2_PO_4_, 4.73 g/L Na_2_HPO_4_, 171 g/L sucrose, pH 7.0) and resuspended in fresh PS buffer. The suspension was incubated with egg white lysozyme (FUJIFILM Wako Pure Chemical, Osaka, Japan) dissolved in PS buffer with 2 mM disodium EDTA (Dojindo, Kumamoto, Japan), at a final concentration of 2 mg/ml. The mixture was incubated at 37 °C for 6 h while shaking gently.

Spheroplasts were centrifuged at 8,000 rpm (4,900 *g*) for 5 min and resuspended in either MMB0 (5 g/L peptone, 1 g/L yeast extract, 0.1 g/L ferric citrate [Sigma-Aldrich, St. Louis, MO, USA]) containing 300 µg/ml penicillin G [Serva, München] or MMB0 containing penicillin G with different concentrations of CaCl_2_ [7]. Penicillin G was added to inhibit regeneration of cell walls in spheroplasts.

### 2.2. Disruption of rodZ homolog in D. grandis

Gene disruption targeting *D. grandis* *rodZ* was performed using a method that was originally developed to generate deletion mutants in *Deinococcus radiodurans* [20], with modifications. A 769-bp DNA fragment upstream of the *rodZ* promoter region and a 770-bp DNA fragment downstream of the *rodZ* open reading frame were amplified via PCR using *D. grandis* genomic DNA and oligonucleotide primer sets (Table 1). For PCR reaction, Tks Gflex DNA polymerase (Takara Bio, Shiga, Japan) was used. A 1,289-bp DNA fragment (KatHPH cassette) containing the *D. radiodurans katA* promoter and the *E. coli* hygromycin-resistance gene (*hph*) from pKatHPH4 [21] was also amplified by PCR using the oligonucleotide primer set pKat-FP and pKatRP (Table 1). The 3 DNA fragments were digested with 4 kinds of FastDigest restriction enzymes (*Kpn*I, *Hin*dIII, *Bam*HI, and *Sal*I) in FastDigest Buffer (Thermo Fisher Scientific, Waltham, MA, USA), and ligated to the *Kpn*I-*Sal*I sites of the pUC19 vector (Takara Bio) to yield a plasmid, pAYA1, carrying the Δ*rodZ*::*hph* mutation. A 2,752-bp DNA fragment containing the Δ*rodZ*::*hph* mutation was amplified from pAYA1 via PCR using the pKat-FP/pKatRP oligonucleotide primer set and introduced into the *D. grandis* wild-type genome.

Transformation of *D. grandis* was performed as follows. *D. grandis* cells (1 ml) cultured at 30 °C for 24 h were washed with 1 ml of TGY broth and resuspended in 0.1 ml of TGY broth. The cell suspension was mixed with 40 µl of 0.3 M CaCl_2_. A 30 µl aliquot of the cell suspension was mixed with 5 µl of DNA and incubated at 30 °C. After 90 min, 2 ml of TGY broth was added to the mixture and cultured at 30 °C. Following 24 h, cells were harvested via centrifugation and resuspended in 0.4 ml of TGY broth. Aliquots of 0.1 ml were spread on TGY agar plates supplemented with 50 µg/ml hygromycin B (FUJIFILM Wako Pure Chemical) and incubated for 2 to 3 d until colonies of transformants appeared on the plate. A single colony was diluted and spread again on TGY agar plate supplemented with 50 µg/ml hygromycin B for pure culture. The resultant strain was designated Δ*rodZ.*

The *D. grandis* genomic DNA was isolated using a FastDNA Spin Kit with a FastPrep-24 Instrument (MP Biomedicals, Santa Ana, CA, USA). Gene disruption was confirmed by amplifying the target allele by genomic PCR using the oligonucleotide primer set HpH-FP and HpH-RP (Table 1).

### 2.3. Complementation test

To perform complementation studies, the shuttle vector pZT29 between *E. coli* and *D. grandis* was used [22]. It has a replication initiator gene, *rep*, from the small latent plasmid pUE30 from *Deinococcus radiopugnans* and a chloramphenicol resistance gene, *cat*, from *E. coli* under the control of the catalase gene promoter kat-p from *D. radiodurans*. A 1,245-bp DNA fragment containing the *rodZ* open reading frame and its promoter region of *D. grandis* was amplified by PCR using the genomic DNA of *D. grandis* and an oligonucleotide primer set (Table 1). Tks Gflex DNA polymerase (Takara Bio) was used for PCR reaction. The DNA fragment and pZT29 were digested with two restriction enzymes (*Eco*RV, *Xho*I [Roche,Diagnostics, Indianapolis, USA]) and ligated into the *Eco*RV-*Xho*I site of pZT29 vector. The plasmid pZT-*rodZ* was generated to express *rodZ*. Then, it was introduced into *D. grandis* Δ*rodZ* :: *hph* mutant.

**Table 1.** Oligonucleotide primers used in this study.

Name	Sequence (5′–3′)	Usage
Dgra-rodZ-Kpn5F^a^	AGCCGGTACCGCTGGTCGGCGGCCTG	Upstream region
Dgra-rodZ-Hind5R^b^	GCATAAGCTTGACCCCGTTACGCTCCTCCT	Upstream region
Dgra-rodZ-Bam3F^c^	CACCGGATCCGGGTGTGAGGACACCCTCCG	Downstream region
Dgra-rodZ-Sal3R^d^	CGCGGTCGACGATCAGCAGCACCTGCCCG	Downstream region
Dgra-rodZ-EcoRV^e^	TCATGATATCCGGGCGTGGAGTTGGCAACATGA	Upstream region
Dgra-rodZ-XhoI^f^	TAGCTCGAGTCAGAAGGTGCGGGTCACGACC	Downstream region
pKat-FP	CGACGGCCAGTGAATTCGAGC	PCR of plasmids
pKat-RP	CAGCTATGACCATGATTACGCCAAGC	PCR of plasmids
Hph-FP	GAGCGAGGAGGAGCGTAAC	Diagnostic PCR
Hph-RP	CACTCTGCTCGATTCACACG	Diagnostic PCR

^a^ *Kpn*I site was underlined.

^b^ *Hin*dIII site was underlined.

^c^ *Bam*HI site was underlined.

^d^ *Sal*I site was underlined.

^e^ *Eco*RV site was underlined.

^f^ *Xho*I site was underlined.

Transformation of *D. grandis* Δ*rodZ* was performed as follows. *D. grandis* Δ*rodZ* cells (1 ml) cultured at 30 °C for 24 h were washed with 1 ml of TGY broth and resuspended in 0.1 ml of TGY broth. The cell suspension was mixed with 40 µl of 0.3 M CaCl_2_. A 30 µl aliquot of the cell suspension was mixed with 0.5 ng of plasmid pZT-*rodZ* and cultured at 25 °C. After 24 h, 2 ml of TGY broth was added to the mixture and cultured at 25 °C. Following 24 h, cells were harvested via centrifugation and resuspended in 0.2 ml of TGY broth. Aliquots of 0.1 ml were spread on TGY agar plates supplemented with 3 µg/ml chloramphenicol (Nacalai,Tesque, Kyoto, Japan) and incubated for 2 days until colonies of transformants appeared on the plate. The resultant strain was designated Δ*rodZ* pZT-*rodZ*.

### 2.4. DAPI staining

To acquire fluorescence microscopy images of nucleoids in enlarged *D. grandis* cells, cell suspension was mixed with 4′,6-diamidino-2-phenylindole (DAPI) (Dojindo) solution to produce a final concentration of 0.5 µg/ml and incubated at 24 °C for 1 h. Bright field, phase contrast, and fluorescence microscopy images were captured using a Keyence BZ-X710 microscope (Osaka, Japan).

### 2.5. Magnesium ion staining

To determine cytoplasm, we stained cytoplasmic Mg^2+^ in *D. grandis* spheroplasts. Cell suspensions were mixed with Magnesium Green, AM cell permeant (Thermo Fisher Scientific) to final concentration of 2 µM, and then incubated in each medium at 24 °C for 10 min. The membrane permeability of this Mg^2+^ indicator dye is enhanced by its acetoxymethyl (AM) ester. After the indicator dye crosses the plasma membrane, non-specific cytoplasmic esterase cleaves the AM ester. Following cleavage, the indicator dye can then bind to Mg^2+^. Phase contrast and fluorescence microscopy images were captured using an Olympus BX51 microscope.

### 2.6. Membrane staining

To acquire fluorescence microscopy images of the membrane in *D. grandis* spheroplasts, the spheroplasts were mixed with FM4-64 (Thermo Fisher Scientific) and DAPI at final concentrations of 5.0 and 0.5 µM, respectively, and incubated for 10 min at room temperature. Phase contrast and fluorescence microscopy images were captured using an Olympus BX51 microscope (Tokyo, Japan).

### 2.7. Cell size measurement

Phase contrast microscopy images of spheroplasts were obtained using an Olympus CK X41 (Tokyo, Japan) or a Keyence BZ-X710 microscope. Cell diameters were measured using CellSens Standard imaging software, version 1.11 (Olympus).

## 3. Results and discussion

**Figure 1.**
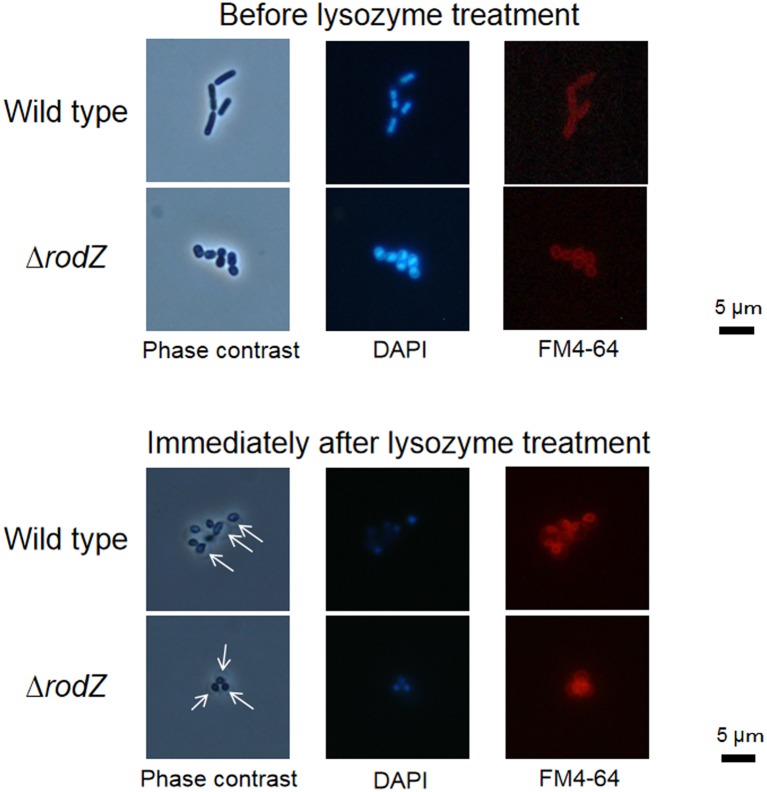
Micrographs of *D. grandis* cells before and immediately after lysozyme treatment. *D. grandis* cells were mixed with FM4-64 and DAPI at final concentrations of 5.0 and 0.5 µM, respectively, and incubated for 10 min. Phase contrast and fluorescence micrographs were captured using an Olympus BX51 microscope. Arrows indicate periplasmic spaces.

We replaced *rodZ* with *hph* using homologous recombination. As *hph* contains a *Pst*I site and *rodZ* does not, we confirmed the complete replacement of *rodZ* by cleaving the PCR product with *Pst*I (Figure S1). Microscopic observation showed that Δ*rodZ* cells were spherical in shape (Figure 1). This result is consistent with the cell shape of *rodZ*-deletion mutants of *E. coli* [14],[15]. Thus, the rod shape of *D. grandis* may also be maintained by the MreB-RodZ system. Although the growth of Δ*rodZ* was slightly slower than that of wild type (Figure S2), cell division in Δ*rodZ* was equal to that of wild type.

The morphology of wild type and Δ*rodZ* spheroplasts immediately following lysozyme treatment were similar (Figure 1). DAPI staining was performed to identify cytoplasm, while FM4-64 staining was performed to identify the membrane. The spheroplasts of both wild type and Δ*rodZ* had a large periplasmic space, while the outer membrane was dissociated from the plasma membrane (Figures 1 and S3).

**Figure 2.**
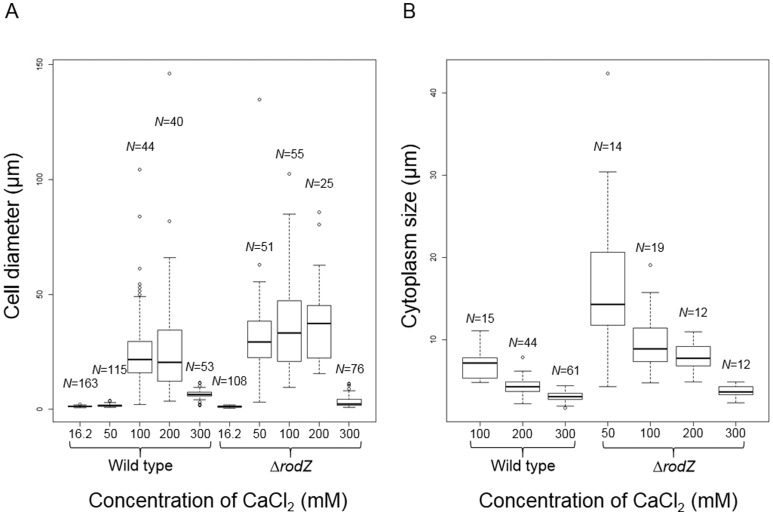
Boxplots of cell diameters and cytoplasm diameters.The sizes measured in this study are shown (Figure S3). The results of statistical tests (pairwise Wilcoxon rank sum test) are shown (Tables S1 and S2).

**Figure 3.**
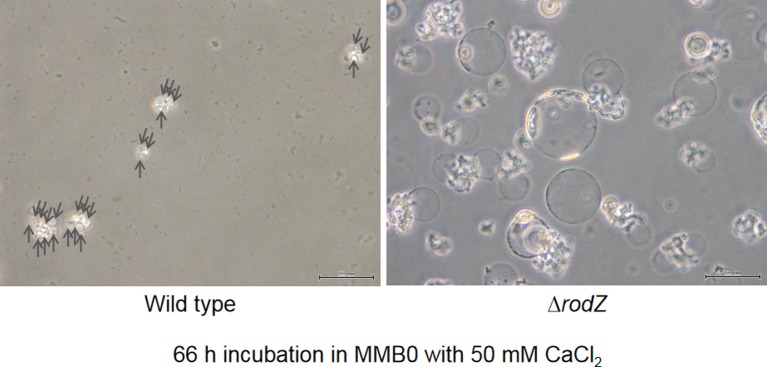
Micrographs of Δ*rodZ* and wild type incubated in MMB0 containing penicillin G with 50 mM CaCl_2_. Phase contrast microscopy images were captured using an Olympus CK X41. The scale bar represents 50 µm. Arrows indicate wild type cells.

Spheroplasts were incubated in MMB0 medium [7] containing penicillin G and different concentration of calcium chloride to generate enlarged cells. The results indicated that Δ*rodZ* were enlarged in 50 mM calcium chloride, whereas wild type was not (Figures 2a, 3 and S4). In addition, although the growth of Δ*rodZ* was slightly slower than that of wild type in the cells with cell walls (Figure S2), cell size of Δ*rodZ* tended to be larger than that of the wild type (Figure 2a; Table S1). This result demonstrated that the Δ*rodZ* spheroplasts displayed a higher sensitivity to calcium ions and enlarged more than the wild-type spheroplasts. The fact that spheroplast enlargement of *D. grandis* required calcium ions [7], implies that the spheroplasts with higher sensitivity to calcium ions tend to enlarge. The biosynthetic speed of the outer membrane formation was higher than that of the plasma membrane during Δ*rodZ* enlargement (Figures 2 and S4). This suggests that *rodZ* disruption may enhance outer membrane expansion in *D. grandis* spheroplasts. Phenotype of Δ*rodZ* spheroplasts containing pZT-*rodZ* was similar to that of wild type (Figure S5). Thus, Δ*rodZ* pZT-*rodZ* was enlarged in 100 mM calcium chloride, whereas 50 mM calcium chloride was not (Figure S5).

The size of outer membrane can be measured, but the size of the cytoplasm cannot be measured in *D. grandis* spheroplasts. This is because a part of the cytoplasm is attached to the outer membrane [7]–[9]. Therefore, in this study, we measured the length between the edges of cytoplasmic areas attached to the outer membrane (Figure S6). It is uncertain whether the area of cytoplasm attached to the outer membrane reflects the size of cytoplasm. However, the size between both edges of the cytoplasm reflects a morphological change in the cytoplasm. The cytoplasm size of Δ*rodZ* was significantly higher (*p* < 0.05) than that of the wild type at 200 mM CaCl_2_ (Figure 2b, Table S2), suggesting that *rodZ* disruption enhanced plasma membrane expansion in *D. grandis* spheroplasts. However, the size measured in this study did not have a corresponding peak at 100 to 200 mM in Δ*rodZ* spheroplasts (Figure 2b), which differed from the enlarged pattern of the outer membrane expansion. As the concentration of CaCl_2_ increased, the cytoplasm size tended to decrease in the spheroplasts of both wild type and Δ*rodZ* (Figure 2b). In terms of cell sizes between in 100 mM and 200 mM of CaCl_2_ (Figure 2a), they were not significantly different in both the wild type and Δ*rodZ* (*p* > 0.05, Table S1). On the other hand, when compared cytoplasm sizes between in 100 mM and 200 mM of CaCl_2_ (Figure 2b), they were not significantly different in Δ*rodZ* but they were significantly different (*p* < 0.05) in the wild type (Table S2). The cytoplasm size significantly decreased in wild type but it did not in Δ*rodZ*. Thus, *rodZ* disruption affects plasma membrane expansion in *D. grandis* spheroplasts.

High concentration (300 mM) of CaCl_2_ inhibited the enlargement (Figure 2), which is consistent with the previous studies [7],[8].

RodZ is located on the plasma membrane. Therefore, it may be expected that Δ*rodZ* tends to have a larger plasma membrane during spheroplast enlargement than that of the wild type. However, the reason why Δ*rodZ* tends to have a larger outer membrane remains unclear.

Considering that outer membrane components are transported across the plasma membrane and through the periplasm to assemble in the outer membrane [13], removal of RodZ from the plasma membrane may affect the transport of outer membrane components.




